# COVID-19: animals, veterinary and zoonotic links

**DOI:** 10.1080/01652176.2020.1766725

**Published:** 2020-05-25

**Authors:** Ruchi Tiwari, Kuldeep Dhama, Khan Sharun, Mohd. Iqbal Yatoo, Yashpal Singh Malik, Rajendra Singh, Izabela Michalak, Ranjit Sah, D. Katterine Bonilla-Aldana, Alfonso J Rodriguez-Morales

**Affiliations:** a Department of Veterinary Microbiology and Immunology, College of Veterinary Sciences, UP Pt. Deen Dayal Upadhayay Pashu Chikitsa Vigyan Vishwavidyalay Evum Go-Anusandhan Sansthan (DUVASU), Mathura, India; b Division of Pathology, ICAR-Indian Veterinary Research Institute, Izatnagar, Bareilly, Uttar Pradesh, India; c Division of Surgery, ICAR-Indian Veterinary Research Institute, Izatnagar, Bareilly, Uttar Pradesh, India; d Sher-E, Kashmir University of Agricultural Sciences and Technology of Kashmir, Shalimar, Srinagar, Jammu and Kashmir, India; e Division of Biological Standardization, ICAR-Indian Veterinary Research Institute, Izatnagar, Bareilly, Uttar Pradesh, India; f Faculty of Chemistry, Department of Advanced Material Technologies, Wrocław University of Science and Technology, Wrocław, Poland; g Department of Microbiology, Tribhuvan University Teaching Hospital, Institute of Medicine, Kathmandu, Nepal; h Semillero de Zoonosis, Grupo de Investigación BIOECOS, Fundación Universitaria Autónoma de las Américas, Sede Pereira, Pereira, Risaralda, Colombia; i Public Health and Infection Research Group, Faculty of Health Sciences, Universidad Tecnologica de Pereira, Pereira, Colombia; j Grupo de Investigacion Biomedicina, Faculty of Medicine, Fundacion Universitaria Autonoma de las Americas, Pereira, Risaralda, Colombia

**Keywords:** COVID-19, SARS-CoV-2, animals, veterinary, zoonosis, transmission, one health

## Abstract

Coronavirus disease 2019 (COVID-19), has spread over 210 countries and territories beyond China shortly. On February 29, 2020, the World Health Organization (WHO) denoted it in a high-risk category, and on March 11, 2020, this virus was designated pandemic, after its declaration being a Public Health International Emergency on January 30, 2020. World over high efforts are being made to counter and contain this virus. The COVID-19 outbreak once again proves the potential of the animal-human interface to act as the primary source of emerging zoonotic diseases. Even though the circumstantial evidence suggests the possibility of an initial zoonotic emergence, it is too early to confirm the role of intermediate hosts such as snakes, pangolins, turtles, and other wild animals in the origin of SARS-CoV-2, in addition to bats, the natural hosts of multiple coronaviruses such as SARS-CoV and MERS-CoV. The lessons learned from past episodes of MERS-CoV and SARS-CoV are being exploited to retort this virus. Best efforts are being taken up by worldwide nations to implement effective diagnosis, strict vigilance, heightened surveillance, and monitoring, along with adopting appropriate preventive and control strategies. Identifying the possible zoonotic emergence and the exact mechanism responsible for its initial transmission will help us to design and implement appropriate preventive barriers against the further transmission of SARS-CoV-2. This review discusses in brief about the COVID-19/SARS-CoV-2 with a particular focus on the role of animals, the veterinary and associated zoonotic links along with prevention and control strategies based on One-health approaches.

## 1. Introduction

In the early days of December 2019, where people planned to welcome New Year 2020, as well as the Chinese New Year, on January 25, 2020, news channels reported suffering of people with sporadic and clustered incidences of “pneumonia of unknown origin” in the city of Wuhan under Hubei province, China (Gao 2020; Lu et al. 2020a). Subsequently, after a month of the first report of infection on December 12, 2019, the causative agent was swiftly identified as a member of *Coronaviridae* family, and on January 12, 2020, the World Health Organization (WHO) designated this fast-spreading virus as “2019-novel coronavirus (2019-nCoV)”, and Novel Coronaviral Pneumonia and CoV-associated diseases were referred to as “COVID-19” by WHO on February 11, 2020 (Du et al. 2020; Gralinski and Menachery 2020). Later, this emerging virus was designated as “SARS-CoV-2” by the Coronavirus Study Group (CSG) of the International Committee on Taxonomy of Viruses (ICTV) (Gorbalenya et al. 2020). On March 11, 2020 the WHO declared the situation as a pandemic which is threatening mankind to a great extent (Chatterjee et al. 2020; Zheng 2020; Phadke and Saunik 2020; Rundle et al. 2020). As of now, SARS-CoV-2 is considered as the seventh coronavirus that infects humans. The other coronaviruses (CoVs) include HKU1, NL63, OC43, 229E, SARS-CoV, and MERS-CoV. Among which SARS-CoV and MERS-CoV are zoonotic and have resulted in high mortality outbreaks in the last two decades, while the others are usually associated with mild upper-respiratory tract illnesses (Wei et al. 2020), and sometimes leading to complicated disease, when occurring in immunocompromised individuals (Villamil-Gómez et al. 2020).

The culinary habits of Chinese people involve the consumption of wild animal meat. The common motivation that is responsible for the human consumption of wild animal meat in China is due to their believed medicinal value as well as the health-promoting effects associated with the consumption of certain wild game animal meats and their products (Harypursat and Chen 2020). The circumstantial evidence that links the first case of COVID-19 to the Huanan South Seafood Market that sells various exotic live animals and our previous knowledge that coronaviruses are animal-derived made us conclude the possible zoonotic transmission in SARS-CoV-2. Nevertheless, it is too early to jump into conclusions since our knowledge of the primary source of infection is limited (Jalava 2020). Identifying the origin of SARS-CoV-2 will help us to unravel the exact mechanism responsible for its initial transmission. After attaining a remarkable progress in developing field oriented as well as high accuracy lab-based diagnostics, much attention has been paved upon developing effective vaccine and therapeutics for blocking person-person transmission, old age infections and health-care workers infection (Chen et al. 2020). That is critical for developing appropriate preventive and control strategies against the fast-spreading SARS-CoV-2 infection. Looking at the beneficial propositions of hydroxychloroquine a multicentric randomised study is underway to assess its effectiveness as a prophylactic measure in curbing secondary SARS-CoV-2 infections as well as associated clinical symptoms progression reducing overall the spread of the virus (Mitjà and Clotet 2020)

Originating from the central part of China, the SARS-CoV-2 pandemic not only dispersed in 369 other cities of China but also crossed the international boundaries within a short period (December to March 2020). As of May 2, 2020, COVID-2019 has affected persons in more than 210 countries and territories in Asia, Europe, Africa, North America, and Latin America (Rodríguez-Morales et al. 2020a; WHO 2020). Hence, due to very high transmissibility across the borders, it was declared as public health emergency of international concern by the WHO on January 30, 2020, and later as pandemic situation (Du Toit 2020; Habibzadeh and Stoneman 2020; Liu et al. 2020a, 2020b; Wood 2020; WHO 2020).

At the beginning of 21^st^ century, other coronaviruses like SARS-CoV and MERS-CoV, in 2002 and 2012, respectively have also caused severe acute respiratory distress (SARD) in the form of outbreaks but the current SARS-CoV-2 pandemic affected wider population accounting a total number of nearly 4.71 million confirmed cases along with death toll of nearly 0.31 million by May 17, 2020 (WHO 2020). These numbers are comparatively higher than SARS-CoV and MERS-CoV cases but with lower case fatality rate. The occurrence of this pandemic has adversely affected the global economy, especially in developing nations. This outbreak not only dejected the multinational businesses, disrupted the global market trading, tourism, transportation, export-import, but also reduced the income generated from the market (Ayittey et al. 2020).

China is home for several farms that rears several animal species such as deer, snakes, porcupines, foxes, civets, bears, turtles, bamboo rats, mink, and birds. Such farms can be targeted to find the origin of SARS-CoV-2 (Zhai et al. 2020). Before declaring snakes, pangolins or even dogs as the reservoir host of SARS-CoV-2, a set of established principles called as the Koch’s postulates have to be satisfied. Hence, it is unethical to cull these animals without any conclusive evidence of SARS-CoV-2 transmission from animals-to-humans (Brownlie 2020). The recent reports of SARS-CoV-2 in animals such as dogs, cats, and a tiger have resulted in unnecessary fear among the general public as well as pet owners and have negatively impacted the welfare of animals (Parry 2020).

The present compilation highlights, in brief, abut SARS-CoV-2, causing emerging coronavirus disease (COVID-19) in humans with regards to the role of animals, veterinary importance, zoonotic aspects, and salient prevention and control strategies focusing on One-health approaches to restrain and combat this pandemic virus.

## 2. The virus (SARS-CoV-2)

Coronaviruses are positive-sense RNA viruses. The newly identified SARS-CoV-2 (2019-nCoV) is a member of the order *Nidovirales,* family *Coronaviridae*, sub-family *Orthocoronavirinae* under which four genera, namely, *Alphacoronavirus, Betacoronavirus, Gammacoronavirus,* and *Deltacoronavirus,* are categorized. The SARS-CoV-2 belongs to the genus *Betacoronavirus* and subgenus *Sarbecovirus.* SARS-CoV and MERS-CoV were also part of the *Betacoronavirus* genus, but SARS-CoV-2 is different from these two at genetic level. The SARS-CoV-2 has been found 88-89% identical to two bat origin SARS coronaviruses (bat-SL-CoVZC45 and bat-SL-CoVZXC21, also named as ZC45 and ZXC21), while it is 82% identical to human SARS-CoV Tor2 and human SARS-CoV BJ01 2003 at the nucleotide level (Drexler et al. 2014; Hu et al. 2017; Hu et al. 2018; Chan et al. 2020; Malik et al. 2020a). Only 50-51.8% identity was observed between SARS-CoV-2 and MERS-CoV and 79% between SARS-CoV-2 and SARS-CoV; further molecular level phylogenetic analyses reveal that SARS-CoV-2 is more close to bat origin SARS-CoV (Mohd et al. 2016; Ramadan and Shaib 2019; Ren et al. 2020; Malik et al. 2020a). The advanced, in-depth genome analysis identified the presence of 380 amino acid substitutions between the sequences of SARS-CoV-2 (HB01) in comparison to the corresponding consensus sequences of SARS-CoV and SARS-CoV like viruses. This amino acid substitution might have contributed to the functional as well as the pathogenic divergence of this novel virus (Wu et al. 2020a).

## 3. Host range

Coronaviruses (CoVs) infect man as well as domestic and wild animal species and usually infections remain sub-clinical in most cases (Ji et al. 2020a; Li et al. 2020a; Salata et al. 2020). The clinical form varies from enteritis in cattle, horses and swine, upper respiratory tract disease in cattle, dogs, felines, and poultry, and common cold to highly fatal respiratory infections in humans (Dhama et al. 2020a, 2020b). Among the four genera in the *Coronaviridae* family, *Alphacoronavirus* and *Betacoronavirus* usually infect mammals and have probable bat origin, while *Gammacoronavirus* and *Deltacoronavirus* infect birds, fishes, and mammals and are assumed to have swine origin (Woo et al. 2012; Hu et al. 2017; Cui et al. 2019). The genus *Betacoronavirus* possess potential zoonotic pathogens like SARS-CoV and MERS-CoV which have bats as primary host and palm civet cat and dromedary camels as intermediate hosts, respectively (Wang and Eaton 2007; Ar Gouilh et al. 2018; Ramadan and Shaib 2019). Many CoVs have been recovered from birds such as Wigeon coronavirus HKU20, Bulbul coronavirus HKU11, Munia coronavirus HKU13, White-eye coronavirus HKU16, Night-heron coronavirus HKU19 and Common moorhen coronavirus HKU21. The common pig infecting coronaviruses include Porcine Coronavirus HKU15, Transmissible Gastroenteritis Virus (TGEV), Porcine Epidemic Diarrhea Virus (PEDV), and Porcine Hemagglutinating Encephalomyelitis virus (PHEV) which are being reported from many parts of the world (Ma et al., 2008). A list of other animal species also reported harbouring the CoVs such as cattle, horses, swine, dogs, cats, camels, rabbits, rodents, birds, ferrets, mink, bats, snake (such as Chinese cobra and krait), frogs, marmots, hedgehogs (*Erinaceus europaeus*), Malayan or Javan or Sunda pangolin (*Manis javanica*), many other wild animals and their role as carrier/reservoir needs urgent attention (WHO 2003; Dhama et al. 2014a, 2014b, 2020a; Monchatre-Leroy et al. 2017; Ji et al. 2020a; Malik et al., 2020b; Xu 2020).

## 4. Covid-19/SARS-CoV-2: animals, veterinary, zoonotic links and transmission

### 4.1. Coronaviruses affecting animals

As coronaviruses have a broad animal host range, several animal species harbour these pathogens, and only a few of them get a severe infection (Cui et al. 2019; Andersen et al. 2020). Coronaviruses like mouse hepatitis virus, rat sialodacryoadenitis coronavirus, guinea pig coronavirus and rabbit coronaviruses are some important CoVs responsible for hepatitis, enteritis, and respiratory infections in lab animals. Among large animals, bovine coronaviruses (BoCoVs) have zoonotic potential as being isolated from asymptomatic children and also found affecting several domestic and wild ruminants, in which calf diarrhea in neonates, bloody diarrhea in adult cattle and respiratory form of shipping fever in all age groups of cattle are universal implications (Zhang et al. 1994; Suzuki et al. 2020). Feline CoVs affect the respiratory tract, central nervous system, abdominal cavity, and gastrointestinal tract to produce enteritis and infectious peritonitis (Tekes and Thiel 2016). Canine enteric coronavirus of *Alphacoronavirus* and canine respiratory coronavirus of *Betacoronavirus* genera affect the enteric and respiratory tract, respectively (Erles and Brownlie 2008; Licitra et al. 2014). In the poultry industry, infectious bronchitis virus (IBV), member of the genus *Gammacoronavirus* cause extensive economic loss by producing respiratory illness, urinary tract infection, and reproductive problems (Dhama et al. 2014a, 2014b). Swine acute diarrhea syndrome coronavirus (SADS-CoV), a member of genus *Alphacoronavirus,* produces severe enteritis in suckling piglets, causing significant mortality. Upon genomic analysis, SADS-CoV was found 95-96% identical to horseshoe bat origin (*Rhinolophus* sp.) coronavirus and named as HKU2 coronavirus (Wang and Jin 2020). It suggested the possibility of host jumping by a coronavirus from bats to pigs by crossing the species barrier either by genetic recombination or by making changes at the level of the receptor-binding domain (RBD) (Zhou et al. 2018; Yang et al. 2019).

Among different animal species, another novel CoV named SW1 has been identified by using panviral microarray technology in the liver tissue of the captive beluga whale (*Delphinapterus leucas*) (Mihindukulasuriya et al. 2008).

### 4.2. Animals and zoonotic links of SARS-CoV-2

Of note, coronaviruses have crossed the species barrier twice in the past during SARS and MERS outbreaks, and thus SARS-CoV-2 looks to be the outcome of species barrier jumping for the third time. Amongst CoVs, recent zoonotic ones such as SARS-CoV, MERS-CoV, and SARS-CoV-2 gained higher importance due to the severity of disease in humans and their global spread (Rothan and Byrareddy 2020). The emergence of novel CoVs and their wide host range may be due to instability of the replicase enzyme, RNA dependent RNA polymerase, polybasic furin cleavage site, and O-linked glycans, lack of proofreading mechanism, a higher rate of mutations in the RBD of spike gene and genetic recombination (Su et al. 2016; Chen 2020; Patel and Jernigan 2020). Researchers also showed that SARS-CoV and SARS-CoV-2 (2019-nCoV) both use ACE2 as a similar cell entry receptor (Zhou et al. 2020a). Due to the mutation in the RBD region of S gene of CoVs, the host-range get expanded to infect other host species of animals or humans, pathogenicity and transmissibility of virus may further get altered and increased, becoming a matter of global worry (Chen 2020; Patel and Jernigan 2020).

**Figure 1.**
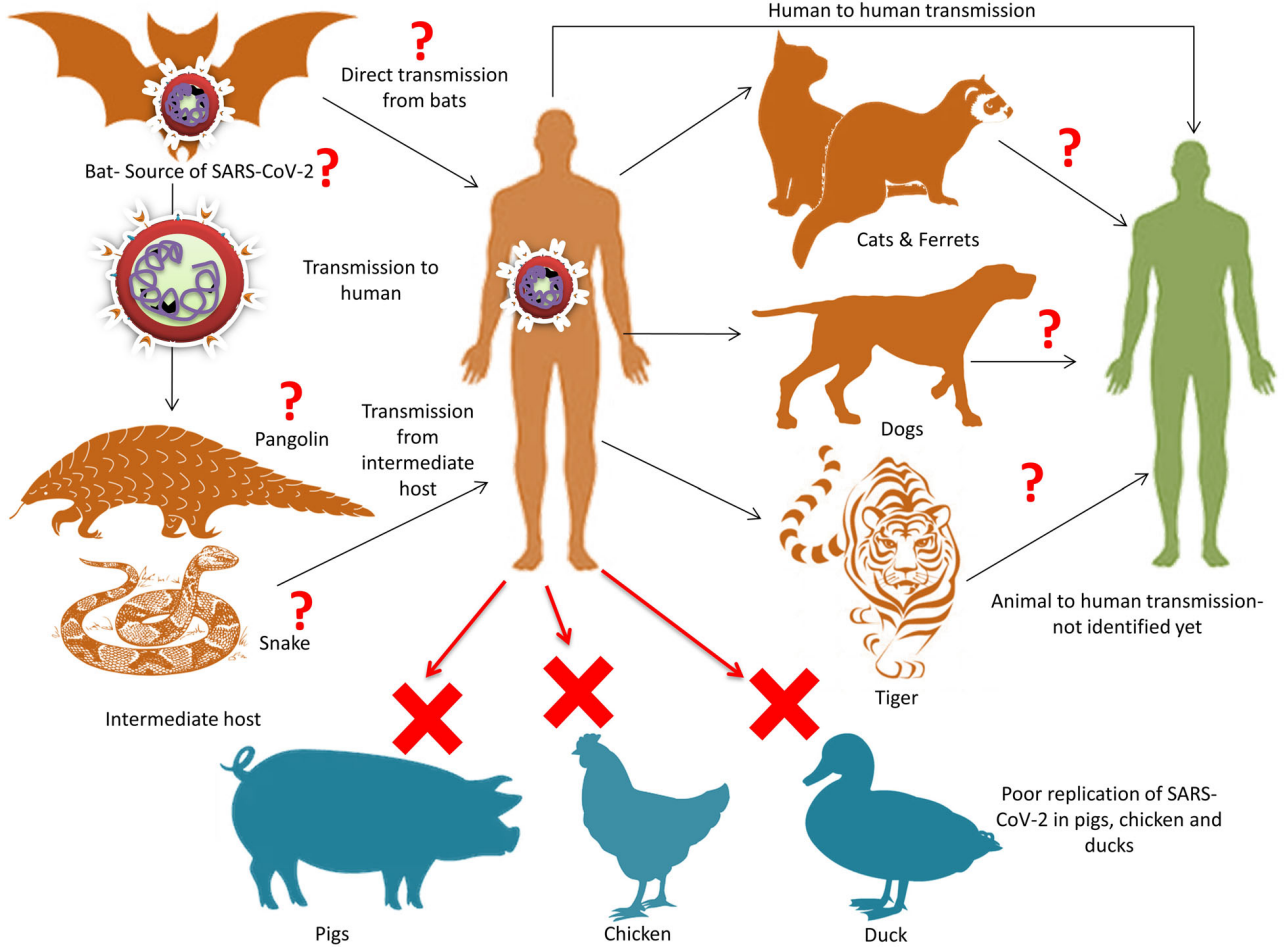
Zoonotic links of SARS-CoV-2. Bat has been reported as the reservoir source of SARS-CoV-2. The intermediate host is not yet elucidated clearly and presently snake and/or pangolins are reported to the intermediate host. Reports regarding the transmission of SARS-CoV-2 from human to animal have been speculated. Study also shows that SARS-CoV-2 replicate poorly in pig, chicken and duck while ferrets and cats are susceptible.

While searching the source of SARS-CoV-2, it was observed that the initially infected individuals had a common exposure spot. It was South China Wet Seafood wholesale market in Wuhan, Hubei Province, China, where restaurants are famous for offering various small and large domestic animals, wild animals, and live animals including poultry, rabbits, bats, snakes, pangolins, turtles, hedgehogs, badgers, and marmots for human consumption (Hu et al. 2015; Hui et al. 2020; Ji et al. 2020b; Liu et al. 2020a, 2020b; Lu et al. 2020b; Wang et al. 2020; Wu et al. 2020b). The initial inferences from Wuhan Seafood Market hypothesised animal source attachments and wild animals for the spillover of SARS-CoV-2. Findings indicated the probability of a zoonotic basis, as CoVs keep on circulating between various animal species, vertebrate, and humans due to a broad host range (Figure 1). It was assumed that SARS-CoV-2 got initially transmitted from animals to humans, followed by maintaining via human-to-human transmission (Hui et al. 2020; Ji et al. 2020a; Nishiura et al. 2020). In the case of MERS-CoV, there is evidence that the viral RNA is not only shed by nasal secretions and feces but also from milk, suggesting the risk of food-borne transmission of MERS-CoV (Reusken et al. 2014). Additionally, a high proportion of camels presenting for slaughter in some studies showed evidence for nasal MERS-CoV shedding (Farag et al. 2015). Further, the possibility of SARS-CoV-2 being the food-borne CoV infection that is transmitted by the respiratory route cannot be rejected (Jalava 2020). Literature documents that a few of the bat origin SARS-CoVs were likely capable of infecting human beings. As seen earlier, bats were found involved in the transmission of SARS-CoV and MERS-CoV, thereby researchers forecasted on the role of bats in the origin and transmission for the current pandemic of SARS-CoV-2 (Fan et al. 2019; Malik et al. 2020a; Wong et al. 2019; Zhou et al. 2020a). For the time being, it is understood that the SARS-CoV-2 is closely related to the bat coronavirus that was isolated from horseshoe bat, the species of bat that is considered to be a maintenance host of previous SARS-related CoVs. Hence, SARS-CoV-2 might have emerged from the sequential recombination occurring between the precursors of SARS-related coronaviruses. Based upon codon usage bias snake SARS-CoV-2 was proposed as the reservoir of SARS-CoV-2 (Ji et al. 2020a). However, later this proposal was contradicted by several researchers. This is the reason for suspecting the presence of an intermediate animal host that is responsible for the zoonotic spill-over to humans (Weiss and Leibowitz 2011; Murdoch and French 2020).

Similarly, not only from bats, but coronavirus associated with SARS was transmitted from human beings to pigs (Chen et al. 2005). It is pertinent to mention that pigs had been predominant species for the evolution of many new strains of Influenza A virus in the past when present in close association with avian and human species and as bat CoVs are infecting pigs, the possibility of evolution of any new virus involving influenza and corona cannot be excluded including the current scenario of growing SARS-CoV-2 cases, such hypothesis needs explorative studies (Brown 2001; Dhama et al. 2012; Malik et al. 2020a). Provided conditions, at any point in time, pigs which serve as a mixing vessel of influenza viruses (Ma et al. 2009) need to be taken with caution as they remain in proximity with man and domestic-sylvatic cycles involving contact with many wild animals and then the situation may get worsen (Ma et al. 2008). However, for the time being, findings of Shi et al. (2020) have not revealed significant susceptibility of pigs to SARS-CoV-2.

Bats, civets, and camels have been the recent animal carriers of human CoV infections (Cui et al. 2019). Of the latest, bats (Wu et al. 2020b) and pangolins (Zhang et al. 2020a) are considered to be the probable sources of origin of SARS-CoV-2 (Andersen et al. 2020). Still, actual intermediate host and nature of emergence are yet to be explored. Two scenarios of the emergence of SARS-CoV-2 are being projected. First is that natural selection of viruses that may have occurred in an animal host before transmission to humans and the second is that natural selection of viruses has occurred in humans after zoonotic transmission (Andersen et al. 2020). Advanced studies involving cell culture or animal models can help in clarifying these hypotheses (Ge et al. 2013; Andersen et al. 2020).

#### 4.2.1. Bats

Bats are ideal reservoir hosts for CoVs, as viruses are persistently present in bats and being asymptomatic. They travel across the forests in search of food and transmit the virus to a variety of hosts they come in contact with (Fan et al. 2019). In China, bats are not only sold for food purposes in live-animal markets, but they are an integral part of Traditional Chinese Medicine (TCM) and wild bats are used to obtain bat-derived compounds. Although bats have commercial value, they pose a severe risk of acquiring any new zoonotic infection (Riccucci 2012; Wassenaar and Zou 2020). In the current COVID-19 pandemic, laboratory findings confirmed that SARS-CoV-2 is also 96% identical to the bat CoV at the genomic level, and hence bats may be the primary source of this zoonotic spillover (Tang et al. 2006; Rodriguez-Morales et al. 2020b; Zhou et al. 2020a).

#### 4.2.2. Pangolins

Not only from bats, coronavirus has been isolated from Malayan Pangolins also, and RBD in S protein of SARS-CoV-2 was nearly the same as that of Pangolin-CoV, and thus pangolins might be the intermediate host of SARS-CoV-2 (Xiao et al. 2020). This is also supported by the findings of Zhang et al. (2020a). Interestingly, the coronavirus isolated from pangolins (SRR10168377 and SRR10168378) did not have the RRAR motif. The SARS-CoV-2 virus isolated from the infected individuals showed higher similarity to the Beta CoV/bat/Yunnan/RaTG13/2013 virus compared to the ones that were isolated from the pangolins (Li et al. 2020b). These findings suggested that the pangolins had less probability of acting as the intermediate host of SARS-CoV-2. Further studies are required to identify the intermediate host and to confirm their role in the origin of SARS-CoV-2 in humans.

#### 4.2.3. Canines and felines

Till now, SARS-CoV-2 infection has been noticed in two dogs; both are reported from Hong Kong (Almendros 2020a). The first case was reported in a 17-year-old Pomeranian dog that gave positive RT-PCR results in both oral and nasal samples (Almendros 2020a; American Veterinary Medical Association 2020). Even though the initial serological test gave a negative result, the blood samples taken in the later stages gave weak positive results. This might be due to the fact that formation of antibody can take 14 days or more (Almendros and Gascoigne 2020b). A report of seroconversion in dogs indicates that the animal has produced antibodies against SARS-CoV-2. This is suggestive of a weak infection in the dog that resulted in an immune response. Hence, the findings are suggestive of a true infection in dogs caused by human-to-animal transmission (Almendros and Gascoigne 2020b). Similarly, another case of SARS-CoV-2 infection was reported in a German Shepherd Dog in Hong Kong. It is interesting to note that both the cases of canine SARS-CoV-2 infections were reported in dogs that were living in close contact with SARS-CoV-2 positive owners (American Veterinary Medical Association 2020). Currently, there is no substantial evidence that dogs get SARS-CoV-2 infection, or can transmit this virus to human beings (Almendros 2020a).

Two cats, one from Belgium and another from Hong Kong, were also tested positive for SARS-CoV-2 (American Veterinary Medical Association 2020). The scientists from Harbin Veterinary Research Institute have reported that cats can get infection with SARS-CoV-2 under experimental conditions and can transmit to other susceptible cats that are housed together (Mallapaty 2020; Shi et al. 2020). The findings are based on experimental inoculation and may not be accurate in natural conditions. None of the infected cats showed any signs of illness, indicating the low potential for transmitting the infection (Mallapaty 2020). A serological study was conducted among the cats of Wuhan that observed the presence of SARS-CoV-2 neutralizing antibodies. This indicates that cats can get SARS-CoV-2 infection under natural conditions resulting in an antibody response (Zhang et al. 2020b). However, among the cats that tested positive, a higher titre of neutralizing antibodies was seen in the cats that were living in close contact with SARS-CoV-2 positive owners (Zhang et al. 2020b). Recently, a Malayan tiger maintained in the Bronx Zoo of New York City, NY, USA was also tested positive for SARS-CoV-2. The “Big cat” is suspected to be infected by SARS-CoV-2 positive asymptomatic zookeeper. These carnivores were tested for SARS-CoV-2 when they started showing signs of mild respiratory illness (United States Department of Agriculture 2020).

### 4.3. Human-animal interactions as a risk factor

Some researchers opined that traditional cooking practices of China are also responsible to some extent for the occurrence of novel CoV infection in humans because as per the Chinese food customs live-slaughtered animals are considered more nutritious, but at the same time, people get exposure to possibly all/any types of pathogens (SARS-CoV, Nipah virus, Hepatitis A virus, Hepatitis E virus, Norovirus, Rotavirus, Highly Pathogenic Avian Influenza virus) present in the offered food items (FAO/WHO 2008). Repeated human-animal interactions either in the market or in the animal industry without using proper environmental biosecurity were considered as the significant risk factors for the emergence of zoonotic diseases, particularly in the rural communities of southern China (Daszak 2020). After these reports, China has put temporarily ban on the sale of wildlife and trading of bats following CoV infection. Furthermore, Wuhan animal food market is also kept closed so that further zoonotic transmission of SARS-CoV-2 and evolution of any new viral variant can be prevented. It is also recommended to avoid any contact with farm or wild animals without the use of personal protective equipment (Benvenuto et al. 2020). Now there is need to draft surveillance strategies and preventive guidelines to have in-depth analysis of bat origin *Betacoronavirus,* especially in the *Rhinolophus* bat family as in the past SARS, MERS, and now SARS-CoV-2 epidemic have created panic, and from epidemic, it has turned to pandemic (Daszak et al. 2020).

In a nutshell, bats appears as the natural reservoir or source of origin for SARS-CoV-2 (Li et al. 2020) that causes zoonotic infection in humans through an intermediate host yet to be deciphered with recent investigations on pangolins, ferrets and possibly snakes. However, the future explorations might reveal the actual intermediate host of SARS-CoV-2 responsible for zoonotic transmission (Almendros 2020a; Dhama et al. 2020d; Shi et al. 2020; Zhang et al. 2020a, 2020b).

## 5. Animal models

Though animal model studies are currently lacking for SARS-CoV-2, a recent study explored the utility of non-human primates, rhesus macaque, as a model for carrying out SARS-CoV-2 studies. Earlier, these non-human primates were used in evaluating the vaccines and antivirals against the MERS-CoV (de Wit et al. 2020). While working on SARS-CoV-2, rhesus macaques showed the establishment of SARS-CoV-2 infection with detection of high virus amount in oral-naso and rectal swabs. The apparent lesions of disease in lung radiographs and clinical signs lasting for up to 16 days proved the effectiveness of the model in studying the pathogenesis of this disease and aiding further in developing and testing vaccines and antivirals (Munster et al. 2020).

Isolation SARS-CoV-2 from dogs is also reported (OIE. 2020). Most recently, Shi et al. (2020) have demonstrated the susceptibility of ferrets, cats, dogs, and different domestic animal species to SARS-CoV-2 by experimental inoculation and reported that SARS-CoV-2 replicates poorly in dogs, pigs, chickens, and ducks, but efficiently in ferrets and cats. Cats can spread infection via droplets (Shi et al. 2020). However, accurate exploration requires specific animal models, especially animals with ACE2 receptors similar to those of humans (Andersen et al. 2020). Establishing suitable animal models will not only help in understanding the disease process but will also help in developing prophylactics and therapeutics (TBRI 2020; Dhama et al. 2020c). Non-human primates are considered to be the appropriate models of human diseases, whereas for exploring etio-pathogenesis of the disease and immune response, other animal models are preferred (TBRI 2020). Non-human primates, mice, and hamsters (Gretebeck and Subbarao 2015) have been used as animal models for SARS and MERS, and some may have the possibility in SARS-CoV-2. Golden Syrian hamsters have been investigated for vaccine protection studies against SARS-CoV strains (Roberts et al. 2008), and suggested to be potential animal model for revealing CoV pathology and pathogenesis along with vaccine efficacy to be tested. Transgenic animals (e.g., mice) have better relevance of simulating SARS-CoV-2 since there are structural differences in ACE 2 receptors in various animal species to which receptor binding domain of spike protein of SARS-CoV-2 binds (Liu et al. 2020b; Wang 2020). On the modeling of ACE2 receptors between various animal species of pigs, ferrets, cats, orangutans, monkeys, bat species, and humans have similar levels of affinity for SARS-CoV-2 based on the structural similarity of their ACE2 receptors (Jarvis 2020). Hence these may have a possible role to be used as animal models with further investigative studies. Small animal models are generally preferred, like mice and rabbits being cheap, easy to manipulate, and ease of availability (Dhama et al. 2020c). Initially, mice appeared to be challenging owing to the difference in receptor ACE2 usage pattern, but transgenic mice are now believed to be relevant models for SARS-CoV-2 (Liu et al. 2020a, 2020b Wang 2020,; Zhou et al. 2020b). Ace2 Knockout Mouse, Tmprss2 Knockout Mouse, Stat1 Knockout Mouse, inbred mice, and Transgenic HLA Mice are being evaluated as animal models for SARS-CoV-2/COVID-19 (Hoffmann et al. 2020; Taconic Biosciences 2020; Wang 2020).

## 6. Prevention and control

The past decade and recent episodes of Zika, Ebola, Nipah, and Bird flu viruses (Munjal et al. 2017; Dhama et al. 2012, 2018; Singh et al. 2019), and lessons learned from previous threats of coronaviruses (SARS- and MERS-CoVs) along with advances in science have paved pace to counter emerging pathogens including the SARS-CoV-2. For this purpose, high efforts are being made to contain and control the spread of this emerging virus haunting the lives of humans and posing even now a pandemic situation. Efforts for rapid diagnosis, strict vigilance, appropriate isolation, and quarantine procedures to halt its further spread, enhanced surveillance and monitoring, strengthening of medical facilities and intensive care units, networking programs, rapid communication and providing updates, knowledge awareness of its public health risks to the general population, high efforts to develop effective vaccines and therapeutics/drugs are being explored optimally. International collaborative efforts and readiness to tackle further heightened emergency to a level of pandemic potential along with following suitable One health approach to combat this emerging virus haunting the lives of billions of human population are being followed effectively (Bonilla-Aldana et al. 2020; Dhama et al. 2013a, 2013b; 2020a, 2020c; Malik et al. 2020a, 2020b; Rodriguez-Morales et al. 2020c). Vaccines appear to be the long-lasting solution for the COVID-19 pandemic. However, currently, there are no vaccines available against it (Shang et al. 2020; Chen et al. 2020). Clues are being taken from the viral structures, pathogenesis, and related coronaviruses (Ahmed et al. 2020; Shang et al. 2020).

Various vaccines are being evaluated by different institutes and companies (Shang et al. 2020), with a few under trials. Moderna, Cambridge, Ma, USA, a biopharma company, started with the mRNA-1273 vaccine in collaboration with CanSino, Hong Kong Special Administrative Region, China (Flanagan 2020). The University of Alabama at Birmingham (UAB), Birmingham, Al, USA, in coordination with Altimmune Inc., Gaithersburg, Md, USA, is developing an intranasal vaccine against COVID-19 and named it as AdCOVID on the pattern of pandemic influenza vaccine and inhalation anthrax vaccine (Hansen et al. 2020). The Clover Biopharmaceuticals company, Chengdu, China, has come out with a SARS-CoV-2 S-protein based subunit vaccine (Clover Biopharmaceuticals 2020).

As of March 20, 2020, WHO has tabulated approximately 44 vaccine candidates targeting SARS-CoV-2, among which few are under clinical evaluation and some under development by various companies and institutions. They included live attenuated, formaldehyde inactivated, protein subunit, DNA, m-RNA, VLP, replicating, and non-replicating vector-based SARS-CoV-2 vaccines. Adenovirus type 5 vector vaccine by CanSino biological Inc., Hong Kong Special Administrative Region, China, and Beijing Institute of Biotechnology, Beijing, China, and LNP-encapsulated mRNA vaccine developed by Moderna/NIAID, Bethesda, Md, USA, is under phase-1 clinical evaluation. Few in pre-clinical stage of clinical evaluation against COVID-19 include DNA plasmid vaccine by Zydus Cadila, Ahmedabad, India, DNA plasmid vaccine through electroporation device by Inovio pharmaceuticals, Plymouth Meeting, Pa, USA, DNA vaccine by Takis/Applied DNA Sciences/Evvivax, Rome, Italy, formaldehyde inactivated alum vaccine by Sinovac, Beijing, China, live attenuated virus vaccine by Codagenix, Farmingdale, New York, USA/Serum Institute of India, Pune, India, MVA encoded VLP by GeoVax/BravoVax, Smyrna, Ga, USA, Ad 26 by Janssen pharmaceutical companies, Beerse, Belgium, ChAdOx1 by university of Oxford, Oxford, UK, adenovirus-based NasoVAX expressing SARS-CoV-2 spike protein by Altimmune, Gaithersburg, Md, USA, Ad5 S (named as GREVAX™) by Greffex, Aurora, Co, USA, oral vaccine by Vaxart, South San Francisco, Ca, USA, Drosophila S2 insect cell expression system using VLPs as protein subunit vaccine by ExpreS2ion, Horsholm, Denmark, S protein based by WRAIR/USAMRIID, Fort Detrick, Md, USA, another S protein by AJ Vaccines, København, Denmark, S-trimer by Clover Biopharmaceuticals Inc., Chengdu, China/GSK, Brentford, UK, peptide based vaccine by Vaxil Bio, Toronto, Ontario, Canada, S protein through baculovirus production system by Sanofi Pasteur company, Swiftwater, Pa, USA, full length S trimers nanoparticle with Matrix M by Novavax, Rockville, Md, USA, gp-96 backbone based vaccine by Heat Biologics, Morrisville, Nc, USA, or University of Miami, Miami, Fl, USA, S1 or RBD protein based by Baylor College of Medicine, Houston, Tx, USA, adjuvanted microsphere peptide vaccine candidate by University of Saskatchewan, Saskatoon, Saskatchewan, Canada, LNP encapsulated mRNA encoding RBD by Fudan University/Shanghai JiaoTong University/RNACure Biopharma, Shanghai, China, sa-RNA (small activating ds-RNA) based COVID-19 vaccine by Imperial College London, London, UK, among others. (https://www.who.int/blueprint/priority-diseases/key-action/novel-coronavirus-landscape-ncov.pdf?ua=1). Though till April 1^st^, 2020 US Food and Drug Administration (FAO) had not announced any confirmed commercial therapeutic or prophylactic vaccine against SARS-CoV-2, nevertheless they have enlisted few potential vaccine candidates which are currently under either preclinical or clinical trials such as mRNA-1273 by Moderna Inc., Bethesda, Md, USA, Inovio’s DNA vaccine INO-4800 against COVID-19 by Inovio Pharmaceuticals, Plymouth Meeting, Pa, USA, along with Ology Bioservices, Alachua, Fl, USA, BNT162 a mRNA vaccine by BioNTech, Mainz, Germany, plant-based COVID-19 vaccine by Medicago, Quebec, Quebec, Canada, oral recombinant COVID-19 vaccine by Vaxart, South San Francisco, Ca, USA, Ii-Key peptide COVID-19 vaccine by Generex Biotechnology, Toronto, Ontario, Canada, among others (Precision Vaccinations 2020). (https://www.precisionvaccinations.com/vaccines/coronavirus-vaccines).

Till the vaccines are developed, alternate disease prevention and control strategies need to be focused on. There is a need for strengthening infrastructure and capacity building with a trained workforce, hospital, health workers to identify the SARS affected patient with the isolation of patients after being doubted for COVID-2019. To apply any prevention, measure the first and foremost step is to diagnose the case with accuracy and speed. While confirming any deadly case, the guidelines of Centers for Disease Control and Prevention (CDC) must be adopted. As the suspected case is a good source of nosocomial spread, the health workers must follow all the precautionary practices while handling the COVID-2019 case. Notably, a facility with negative air pressure is recommended for keeping confirmed patients of SARS-CoV-2.

The applications of telemedicine having tele-visits and supervision, monitoring, interpretation, and consultation (Serper and Volk 2018) have proven effective in mitigating chronic diseases. The tele-model has been applied on present-day COVID-19 pandemic, especially in the remote areas having limited medical backups saving both manpower as well as resources (Au 2020).

Although respiratory tract infections and spread of the virus from naso-oral secretions is well discussed, the first isolation of SARS-CoV-2 in China from a COVID-19 positive patient highlighted the significance of the digestive tract in conjunction to the respiratory tract. Since then, while planning the control strategies, this alternative route of virus spread must be kept in mind, including a focus on clinical sufferers, and asymptomatic patients or individuals having no or mild signs (Gu et al. 2020).

Further spread of SARS-CoV-2 can be controlled by immediate isolation of confirmed cases along with contact tracing. According to the analysis made by using transmission models, the COVID-19 outbreak could be controlled within three months by following active contact tracing in combination with the isolation of infected individuals (Hellewell et al. 2020). It is not the first time that live-animal markets in China have been identified as the epicenter of emerging novel zoonotic diseases. For preventing the likelihood of another zoonotic outbreak, closure of all the live-animal markets is a necessity (Peeri et al. 2020). However, the deep connection existing between these live markets and the Chinese culture makes the permanent ban practically impossible. Due to the possible role played by live-markets in the origin of SARS-CoV-2, a temporary ban was imposed on the wildlife trade in China (Harypursat and Chen 2020; Mallapaty 2020), which was again cancelled merely after 4 months of the outbreak as COVID-19 seemed under control in China. Purchase of live-dead animals like dogs, cats, bats, birds, ostriches, rabbits, hamsters, scorpions, badgers, pangolins (with peculiar scaly anteaters), minks, palm civet, snapping turtles, ducks, fishes, Siamese crocodiles (Knowles 2020) (https://www.dailymail.co.uk/news/article-8163761/Chinese-markets-selling-bats.html) again started in more than hundreds of animal markets without imposing strict hygienic preventive measures, and this may again be a predisposing factor for recurrence of health ailment due to COVID-19 in China (Anonymous 2020) (https://metrosaga.com/wuhan-virus-chinese-animal-markets-reopened-with-almost-no-precautions/). A bold decision on the future of wild animal trade in China is a necessity to prevent possible future outbreaks due to zoonotic spillover and hence International agencies like WHO, FAO and OIE must frame strict guidelines with regulations and conditional penalties on purchase of food animals in markets for safer future (Blakeman 2020)(https://thehill.com/opinion/international/490528-china-must-close-down-wet-markets-now). A total ban on the wild animal trade will affect the livelihood of a substantial proportion of the population. Hence such a move will only shift the trade of wild animals to the black market (Mallapaty 2020).

The significance of rapid sharing of the technical facts during the COVID-19 outbreak situations in minimizing the panic perpetuation in public was assessed recently by Song and Karako (2020). It was pointed out that scientific data sharing further enriches the epidemiologists, healthcare workers, and forecasting models to comprehend the usefulness of available interferences from new real-time information (Song and Karako 2020). Furthemore, Habibi et al. (2020) emphasized the significance of following International Health Regulations (IHR) measures to defeat the spread of SARS-CoV-2 across the globe (Zhang et al., 2020).

## 7. Conclusion and future prospects

The live-animal markets, just like the Huanan South China Seafood Market, will continue to act as an ideal point that promotes inter-species contact between the wild and domestic animal species. Hence, the possibility of inter-species transmission of CoV infections at such hot spots is a point of concern to human beings due to the adaptive genetic recombination that occurs in these viruses. The permanent ban on the wild animal trade should not be implemented as it will only shift the trade to the black market. Rather than going for the complete ban, it is better to regulate the trade of wild animal species all around the country. The emergence of newer zoonotic infections like SARS-CoV-2 is inevitable in the future. Hence, local and international regulatory authorities need to develop and implement robust disease control mechanisms that effectively decrease the possibility of human exposure to wild animals. The SARS-CoV-2 outbreak is just another critical example that proves the existence of a close but straightforward interaction between humans, animals, and the environmental health that can potentially result in the emergence of a deadly pandemic. The past decades have shown us the destructive potential of several zoonotic coronavirus infections like SARS, MERS, and now SARS-CoV-2 that calls for the implementation of One Health as a framework to protect humankind from emerging pathogens soon.
